# Total flavonoids of *Desmodium styracifolium* attenuate nephrolithiasis via reducing calcium oxalate crystallization and renal inflammation

**DOI:** 10.3389/fphar.2026.1873638

**Published:** 2026-09-18

**Authors:** Yang Yang, Yingxue Yang, Jingyu Ouyang, Zhikui Cheng, Zhuojian Lu, Xinyong Lv, Jiacheng Wei, Xin Zhao, Yun Feng, Yang Yue, Wentao Du, Lifei Liu, Xuejun Zhang

**Affiliations:** Hubei Bio-Pharmaceutical Industrial Technological Institute Inc., Humanwell Healthcare (Group) Co, Ltd., Wuhan, Hubei, China

**Keywords:** calcium oxalate crystallization, inflammation, nephrolithiasis, total flavonoids of *Desmodium styracifolium*, urolithiasis

## Abstract

**Introduction:**

*Desmodium styracifolium*, known as Guangjinqiancao in Chinese, is a widely used traditional medicinal herb in Southern China for clearing heat and excreting dampness. It has been historically prescribed for the treatment of urological disorders, particularly urolithiasis (Shi Lin). While the clinical efficacy of *D. styracifolium* is well-documented, the precise mechanisms of its primary active fraction, total flavonoids of *D. styracifolium* (TFDS), regarding crystallization and inflammation remain to be fully elucidated. This study aims to investigate the therapeutic mechanism of TFDS in alleviating nephrolithiasis by systematically evaluating its effects on calcium oxalate (CaOx) crystallization kinetics, crystal-cell interaction, and renal inflammatory responses.

**Methods:**

The inhibitory effects of TFDS on calcium oxalate monohydrate (COM) crystallization were quantified *in vitro*, assessing crystal nucleation and growth. Flow cytometry and microscopy were utilized to evaluate the effect of TFDS on crystal adhesion and endocytosis in renal tubular epithelial cells. RT-qPCR was used to assess the expression of inflammatory cytokines. Furthermore, an ethylene glycol and ammonium chloride-induced rat model of nephrolithiasis was employed to evaluate the anti-lithogenic effect of TFDS *in vivo*, utilizing ultrasonography, serum/urine biochemistry, and histopathological analysis.

**Results:**

*In vitro* assays demonstrated that TFDS significantly inhibits COM crystal formation and growth in a dose-dependent manner. Notably, TFDS promoted the dissolution of pre-formed crystals and effectively blocked the adhesion and internalization of crystals by renal cells. Moreover, TFDS treatment suppressed the crystal-induced upregulation of pro-inflammatory cytokines (IL-6, IL-8, TNF-α). *In vivo* experiments demonstrated that the administration of TFDS significantly attenuated renal crystal retention, reduced tissue damage, and improved renal function.

**Conclusion:**

TFDS exerts a potent anti-lithogenic effect by targeting multiple stages of the crystallization process—inhibiting crystal nucleation and growth, promoting dissolution, and preventing cellular adhesion and endocytosis,and suppressing associated inflammation. These findings provide a scientific cognition for the traditional use of *D. styracifolium* and elucidate the mechanism of action for TFDS-based therapies in managing nephrolithiasis.

## 1 Introduction

Renal calculi, commonly referred to as kidney stones, represent a prevalent urological disorder characterized by the deposition of mineral crystals within the renal parenchyma and urinary tract. Calcium oxalate (CaOx) calculi are the predominant subtype, accounting for approximately 70%–80% of all clinical cases (Evan, 2010; Bargagli et al., 2025). Recent decades have seen a sharp rise in nephrolithiasis cases, making it a major public health issue. It primarily affects working-age adults, the condition imposes a heavy economic and societal burden through high healthcare costs and significant productivity loss (Romero et al., 2010; Ward et al., 2019; Filler et al., 2023; Chen et al., 2024). Despite advancements in surgical interventions, a subset of patients remains unsuitable for operative treatment, such as those with small stone burdens or high perioperative risks. Furthermore, the high recurrence rate of nephrolithiasis underscores the urgent need to develop effective pharmacological strategies for the prevention of stone formation.

*Desmodium styracifolium* is a widely utilized traditional Chinese medicinal herb, predominantly cultivated in the southern regions of China (Ma et al., 2011). It is taxonomically classified within the order *Fabales*, family Fabaceae, and genus *Desmodium* (Opryshko et al., 2024). *Desmodium styracifolium* contains diverse constituents, including flavonoids, alkaloids, polysaccharides, phenolic acids, volatile oils, and multiple inorganic elements (Kubo et al., 1989; Zhao et al., 2007). Among these, flavonoids have been identified as the principal therapeutic agents responsible for the majority of the pharmacological activities. Moreover, accumulating evidence indicates that total flavonoids of *D. styracifolium* (TFDS) exert significant protective effects in the management of urological diseases. In a hydroxyl-L-proline-induced rat urolithiasis model, TFDS effectively reduce the deposition of CaOx crystals in renal tissues (Zhou et al., 2018). Furthermore, TFDS alleviate CaOx crystal-induced renal cell apoptosis and autophagy via the p38 and KIM-1 signaling pathways (Xie et al., 2018). Additionally, TFDS exert a protective effect against renal ischemia-reperfusion injury by inhibiting ferroptosis through the regulation of the P53/SLC7A11/GPX4 signaling axis (Yuan et al., 2025). However, the precise and systemic mechanisms underlying their therapeutic efficacy remain to be fully elucidated.

Developed by Humanwell Healthcare (Group) Co., Ltd., *D. styracifolium* total flavonoids capsules represent the innovative traditional Chinese drug whose therapeutic efficacy against urological calculi has been clinically validated and approved by the National Medical Products Administration (NMPA) in 2022 in China. Clinically, evidence demonstrates that patients receiving this formulation exhibit significantly greater calculi expulsion rates than who receiving placebo (clinical trial registration number: CTR20171264). However, despite its established clinical utility and defined chemical composition, the specific cellular and biophysical mechanisms underlying the anti-lithogenic effects of TFDS remain poorly understood, highlighting a critical gap in current empirical research.

In this study, we provide a novel perspective on the anti-urolithic properties of TFDS, which exert therapeutic effects against nephrolithiasis by inhibiting calcium oxalate crystallization, promoting calculus dissolution, and suppressing inflammation.

## 2 Materials and methods

### 2.1 Cell culture

MDCK renal tubular epithelial cell line derived from distal tubular segment of the nephron (ATCC) was propagated in dulbecco’s modified eagle medium (DMEM) (Gibco, C11995500BT) supplemented with 5% fetal bovine serum (FBS) (Gibco, 10,099-141C). Human kidney proximal tubular epithelial cell line (HK-2 cells) was purchased from Wuhan Pricella Biotechnology and cultured in Minimum Essential Medium (MEM) (Gibco, C11095500BT) supplemented with 10% FBS, 1% non-essential amino acid (Servicebio, G4219-100 mL), 1% penicillin-G/streptomycin (Gibco, 15140122). The cells were maintained in a humidified incubator with 5% CO_2_ at 37 °C.

### 2.2 Calcium oxalate monohydrate (COM) crystallization assay

Each well of a 24-well plate (Corning, 3524) was filled with 500 μL of 2.5 mM CaCl_2_ in crystallization buffer (10 mM Tris-HCl, 90 mM NaCl, pH 7.4). Then, 4 μL of the DMSO (control) or TFDS (60, 120, or 240 μg/mL, obtained from Hubei Bio-PharmaceuticaI Industrial TechnologicaI Institute Inc. was added and mixed. Afterwards, 500 μL of 1.0 mM Na_2_C_2_O_4_ in the same buffer (pH 7.4) was introduced to achieve final concentrations of 1.25 mM CaCl_2_ and 0.5 mM Na_2_C_2_O_4_. The final concentration of DMSO was 0.1% for DMSO control and all different concentrations of TFDS groups. Following incubation at 37 °C for 3 h, crystallization was examined. Images were acquired from at least 10 randomly selected high-power fields (HPFs, 200x magnification) under a research inverted microscope (OLYMPUS, IX73). Crystal size and number were quantified using ImageJ software (Schneider et al., 2012). Briefly, the color threshold was setup as black and white after the scale was set as the actual distance. All the hollow crystals were filled completely. Then the particles were analyzed and the crystal size and number were calculated. Total crystal area was calculated using the following formula:

$$\text{Total crystal area }\left(µm^{2}/\text{HPF}\right)=\text{Average crystal size in each field }\left(µm^{2}\right)\times \text{Number of crystals in each field }\left(/\text{HPF}\right)$$

### 2.3 COM crystal growth assay

Equal volumes (500 µL each) of 2.5 mM CaCl_2_ and 1.0 mM Na_2_C_2_O_4_ in crystallization buffer (10 mM Tris-HCl, 90 mM NaCl, pH 7.4) were combined in the wells of a 24-well plate. The mixture was incubated at 37 °C for 2.5 h to achieve complete crystallization. Then, DMSO (negative control) or TFDS (final concentration: 60, 120, or 240 μg/mL) was added to each well, followed by further incubation for 20 h. The final concentration of DMSO was 0.1% for DMSO control and all different concentrations of TFDS groups. Crystal images were captured randomly from at least 10 high-power fields (HPFs) under OLYMPUS IX73 research inverted microscope. Crystal size and number were measured using ImageJ software. Total crystal area was calculated using the following formula:

$$\text{Total crystal area }\left(µm^{2}/\text{HPF}\right)=\text{Average crystal size in each field }\left(µm^{2}\right)\times \text{Number of crystals in each field }\left(/\text{HPF}\right)$$

### 2.4 COM crystal dissolution assay

Equal volumes (500 µL each) of 2.5 mM CaCl_2_ and 1.0 mM Na_2_C_2_O_4_ in crystallization buffer (10 mM Tris-HCl, 90 mM NaCl, pH 7.4) were combined in each well of a 24-well plate. The mixture was incubated at 37 °C for 3 h to allow complete crystallization. Subsequently, the crystals were washed with physiological saline (pH 7.4), and the medium was replaced with 1 mL of physiological saline per well. For the dissolution assay, 4 µL of DMSO (negative control) or TFDS (60, 120, or 240 μg/mL) was added to each well, followed by further incubation for 20 h. The final concentration of DMSO was 0.1% for DMSO control and all different concentrations of TFDS groups. Crystal images were randomly captured from at least 10 high-power fields (HPFs) using an OLYMPUS IX73 research inverted microscope. Crystal size and number were measured using ImageJ software. Total crystal area was calculated using the following formula:

$$\text{Total crystal area }\left(µm^{2}/\text{HPF}\right)=\text{Average crystal size in each field }\left(µm^{2}\right)\times \text{Number of crystals in each field }\left(/\text{HPF}\right)$$

### 2.5 COM crystal preparation

Equal volumes (20 mL each) of 20 mM CaCl_2_ and 1.0 mM Na_2_C_2_O_4_ in Crystallization Buffer (10 mM Tris-HCl, 90 mM NaCl, pH 7.4) were mixed in a 50 mL conical tube. The solutions were incubated at 25 °C overnight and COM crystals were harvested by centrifugation at 2,000 g for 5 min. After the discard of the supernatant, the crystals were washed three times with methanol, followed by another centrifugation under the same conditions. The methanol was discarded, and the crystals were air-dried overnight at 25 °C. The crystals were sterilized by UV light radiation for 30 min before adding to the cells.

### 2.6 COM crystal cell adhesion assay

COM crystals were generated as mentioned in 2.5. MDCK cells were seeded at a density of 1.5 × 10^5^ cells/mL in a 24-well plate and cultured for 24 h to form a confluent monolayer. After refreshing the culture medium, COM crystals were added, followed by 4 µL of DMSO (control) or TFDS (60, 120, or 240 μg/mL). The final concentration of DMSO was 0.1% for DMSO control and all different concentrations of TFDS groups. Cells were then incubated at 37 °C under 5% CO_2_ for 1 h. Thereafter, the cells were vigorously washed with PBS five times and imaged under an OLYMPUS IX73 research inverted microscope.

### 2.7 COM crystal internalization assay

Preparation of fluorescein isothiocyanate (FITC) labeled COM Crystals: Crystals were prepared as described in Section 2.5, except that 0.2 μg/mL FITC was added to the CaCl_2_ solution (pH 7.4) prior to mixing with sodium oxalate. All subsequent steps, including crystallization and harvesting, were conducted in the dark to prevent photobleaching.

Crystal Internalization Assay: HK-2 cells were seeded in 6-well plates at a density of 1.5 × 10^5^ cells per mL and cultured for 24 h. After a medium change, cells were treated with FITC-labeled COM crystals along with either 8 µL DMSO (control) or TFDS (60, 120, or 240 μg/mL), and then incubated at 37 °C under 5% CO_2_ for 24 h. The final concentration of DMSO was 0.1% for DMSO control and all different concentrations of TFDS groups. To remove non-internalized crystals, cells were washed with PBS and detached using trypsin-EDTA. Internalization was quantified by flow cytometry (Agilent, NovoCyte Advanteon), acquiring 10,000 events per sample. The fluorescence threshold was set using cells incubated with unlabeled crystals, and the percentage of FITC-positive cells was calculated.

### 2.8 COM crystal-induced inflammation assay

HK-2 cells were seeded in 6-well plates at a density of 1 × 10^5^ cells/mL and cultured for 24 h. The medium was then replaced, and cells were treated as follows for an additional 24 h: the control group received 8 µL of DMSO; the COM group was exposed to COM crystals, IL-1β (20 ng/mL) or LPS (10 μg/mL); and the treatment groups received COM crystals, IL-1β (20 ng/mL) or LPS (10 μg/mL) plus TFDS at concentrations of 60, 120, or 240 μg/mL. The final concentration of DMSO was 0.1% for DMSO control and all different concentrations of TFDS groups.

Total RNA was extracted using the VAMNE Magnetic Universal Total RNA Kit (Vazyme, ROA3302-02). Complementary DNA (cDNA) was synthesized with the HiScript III All-in-one RT SuperMix Perfect for qPCR (Vazyme, R333). Quantitative real-time PCR (qPCR) was subsequently performed using Taq Pro Universal SYBR qPCR Master Mix (Vazyme, Q712-02) on a Roche LightCycler 96 system. The expression levels of *IL-6*, *IL-8*, and *TNF-*α were normalized to that of the geometric mean of *Gapdh* and *β-actin*. The standard deviation of either *Gapdh* or *β-actin* was less than 0.3. A list of qPCR primers was shown in Table 1.

**TABLE 1:** qPCR primers.

Name	Sequence (5′-3′)
IL-6 forward primer	ACT​CAC​CTC​TTC​AGA​ACG​AAT​TG
IL-6 reverse primer	CCA​TCT​TTG​GAA​GGT​TCA​GGT​TG
IL-8 forward primer	GAG​AGT​GAT​TGA​GAG​TGG​ACC​AC
IL-8 reverse primer	CAC​AAC​CCT​CTG​CAC​CCA​GTT​T
TNF forward primer	CCA​TCA​GAG​GGC​CTG​TAC​CT
TNF reverse primer	GCT​CTT​GAT​GGC​AGA​GAG​GA
GAPDH forward primer	TCC​AAA​ATC​AAG​TGG​GGC​GA
GAPDH reverse primer	TGA​TGA​CCC​TTT​TGG​CTC​CC
β-actin forward primer	CCA​CCA​TGT​ACC​CTG​GCA​TT
β-actin reverse primer	TCT​TCA​TTG​TGC​TGG​GTG​CC

After the cell lysis for 30 min on ice in RIPA buffer (Solarbio, R0010) with protease inhibitors (MCE, HY-K0010) and phosphatase inhibitors (MCE, HY-K0021), the supernatants were collected by centrifugation for 10 min at 4 °C. A BCA kit (Beyotime, P0009) was used to measure the protein concentration. Following denatured samples, 12% SDS-PAGE gels were loaded, electrophoresis was used to separate the samples, and NC membranes were transferred. The membranes were then blocked with 5% skim milk for an hour at room temperature, after three washes with Tris Buffered Saline with Tween 20 (TBST), then incubated with the primary antibody (anti-P-p65, Cell Signaling Technology, 3033S; anti-p65, proteintech, 10745-1-AP; anti-GAPDH, proteintech, 60004-1-Ig) overnight at 4 °C. Then three washes with TBST, subsequently the membranes were exposed using an ECL kit (Thermo Scientific, 34,577) after being incubated with secondary antibody (Thermo Fisher Scientific, A-21244) solutions at room temperature for 1 hour then six washes with TBST. Membranes were filmed using an imaging system (e-BLOT, Touch imager Xli) and the protein expression levels were quantified by a software (eBLOT15, version 15.1.211,025.1).

### 2.9 Animals and interventions

This study was carried out by the ethical principles established and approved by the Animal Experimentation Ethics Committee (approval no: IACUC- 2024(3)145). The researcher assured that the guidelines proposed by Grundy (2015) were adhered to and consistently followed throughout the study.

42 healthy adult male Sprague-Dawley rats (7 weeks old, weighing 190–220 g) were housed under standard conditions (12 h light/12 h dark cycle, 24 °C ± 1 °C, 50%–55% humidity) with *ad libitum* access to food and water.

The rats were randomly assigned to two groups: a vehicle control group (n = 12) and a model induction group (n = 30). Rats in the induction group received 1% ethylene glycol (E.G.,) in their drinking water during the first week, which was increased to 1.25% in the second week and maintained at 1.5% from weeks 3–4. Concurrently, they were administered 10 mg/kg ammonium chloride via oral gavage daily for 28 days to induce kidney calculi formation. The vehicle control group received same volume of saline.

After the 28-day model induction, rats were redistributed into four groups receiving daily oral gavage for 28 days: Vehicle group (original vehicle rats, n = 12, given deionized water), a Model group (n = 10, given deionized water), a TFDS Low group (200 mg/kg TFDS, n = 10), and a TFDS High group (400 mg/kg TFDS, n = 10).

### 2.10 Sample collection

At the end of the experiment, urine samples were collected from rats placed in metabolic cages (Yuyan, Y-3202) 1 day before the sacrifice. The urine was centrifuged to remove debris and stored at 4 °C for further analysis. Subsequently, animals were weighed and anesthetized with an intraperitoneal injection of 2.5% tribromoethanol. Blood was collected via abdominal aortic puncture; serum was separated and stored at −20 °C for subsequent biochemical assays. The left kidney from each rat was fixed in 4% paraformaldehyde (Servicebio, G1101-500 ML) at room temperature for histopathological examination. The right kidney was homogenized, and the resulting tissue homogenate was aliquoted and stored at −80 °C for further biochemical analysis.

### 2.11 Evaluation of biochemical parameters

Biochemical parameters in urine, including urea nitrogen (Rayto, S03036) and creatinine (Nanjing Jiancheng, C011-2-1), and serum, including urea nitrogen (Rayto, S03036) and creatinine (Rayto, S03035) were measured using commercial assay kits. All procedures followed the manufacturer’s instructions, with urine samples analyzed within 24 h of collection.

### 2.12 Ultrasonography measurement of the kidney

Renal ultrasound examination was performed on day 28. Rats were anesthetized with isoflurane, and the abdominal hair was shaved. After applying an ultrasound coupling gel, the kidneys were visualized using a high-resolution ultrasound system (Mindray, Labus7T). To ensure consistency, all examinations and measurements were conducted by the same operator.

### 2.13 Histopathological evaluation

Tissue Processing and Staining: Kidneys were fixed in 4% paraformaldehyde, paraffin-embedded, and sectioned at 4 μm. Sections were stained with hematoxylin and eosin (H&E) for histological evaluation and with a von Kossa staining kit (Leagene, DS0003) for calcium deposition.

Crystal Identification and Quantification: To confirm the crystalline nature of deposits, von Kossa-stained sections were examined under polarized light using an Olympus BX53 microscope. Crystal formation was quantified from the images using Image-Pro Plus software (Media Cybernetics, version 7.0).

### 2.14 Statistical analysis

Statistical analyses were performed using Prism software (GraphPad, version 8.0). Data are presented as the mean ± standard deviation (SD). Differences between two groups were assessed using ANOVA. A P-value of less than 0.05 was considered statistically significant.

## 3 Results

### 3.1 TFDS inhibits COM crystal formation and reduces total crystal area per HPF

To evaluate the inhibitory potential of TFDS on calcium oxalate monohydrate (COM) crystallization, varying concentrations of TFDS were introduced into the crystallization buffer prior to the crystal formation. COM crystal quantity and size were measured and total crystal area per HPF was calculated. DMSO served as the control. Microscopic analysis revealed that TFDS treatment dramatically reduced both the quantity and size of COM crystals in a dose-dependent manner (Figures 1A–C). Consequently, the total crystal area per HPF was significantly lower in the TFDS-treated groups compared to the control (Figure 1D), indicating a potent inhibitory effect on the initial formation of crystals.

**FIGURE 1:**
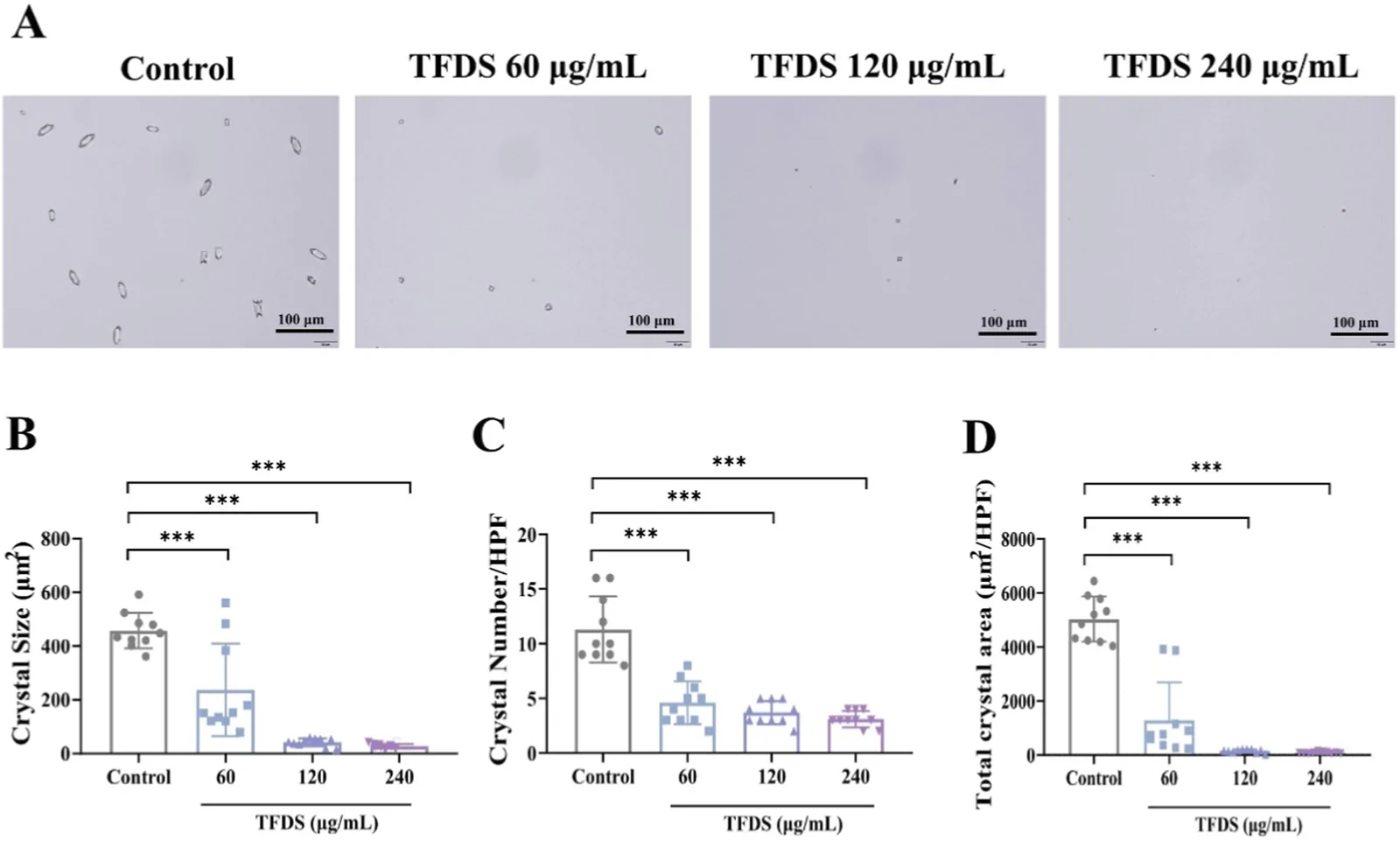
Inhibition of COM crystal formation by TFDS. Crystallization was induced in the presence of DMSO (control) or TFDS (60–240 μg/mL). **(A)** Representative images of crystal morphology under each condition after 3 h. Scale bar, 100 µm. **(B)** Quantification of crystal size. **(C)** Quantification of crystal number. **(D)** Calculated total crystal area per HPF (Formula 1, Materials and Methods). Data are presented as mean ± SD. *p < 0.05, **p < 0.01, ***p < 0.001.

### 3.2 TFDS inhibits the growth of COM crystals

To further examine the influence of TFDS on the growth of COM crystals, graded concentrations of TFDS were introduced into the crystallization buffer following the formation of initial crystallization. The resultant COM crystals were subsequently assessed through microscopic observation after 20 h incubation with TFDS or DMSO, which served as the control (Figure 2A). Both of the size and mass of crystals significantly decreased when increasing the concentration of TFDS (Figures 2B,D). However, the quantity of crystals did not significantly change after adding TFDS (Figure 2C). Taken together, these data indicate that TFDS inhibits the growth of the crystals by reducing the size and mass but not the quantity after COM crystallization.

**FIGURE 2:**
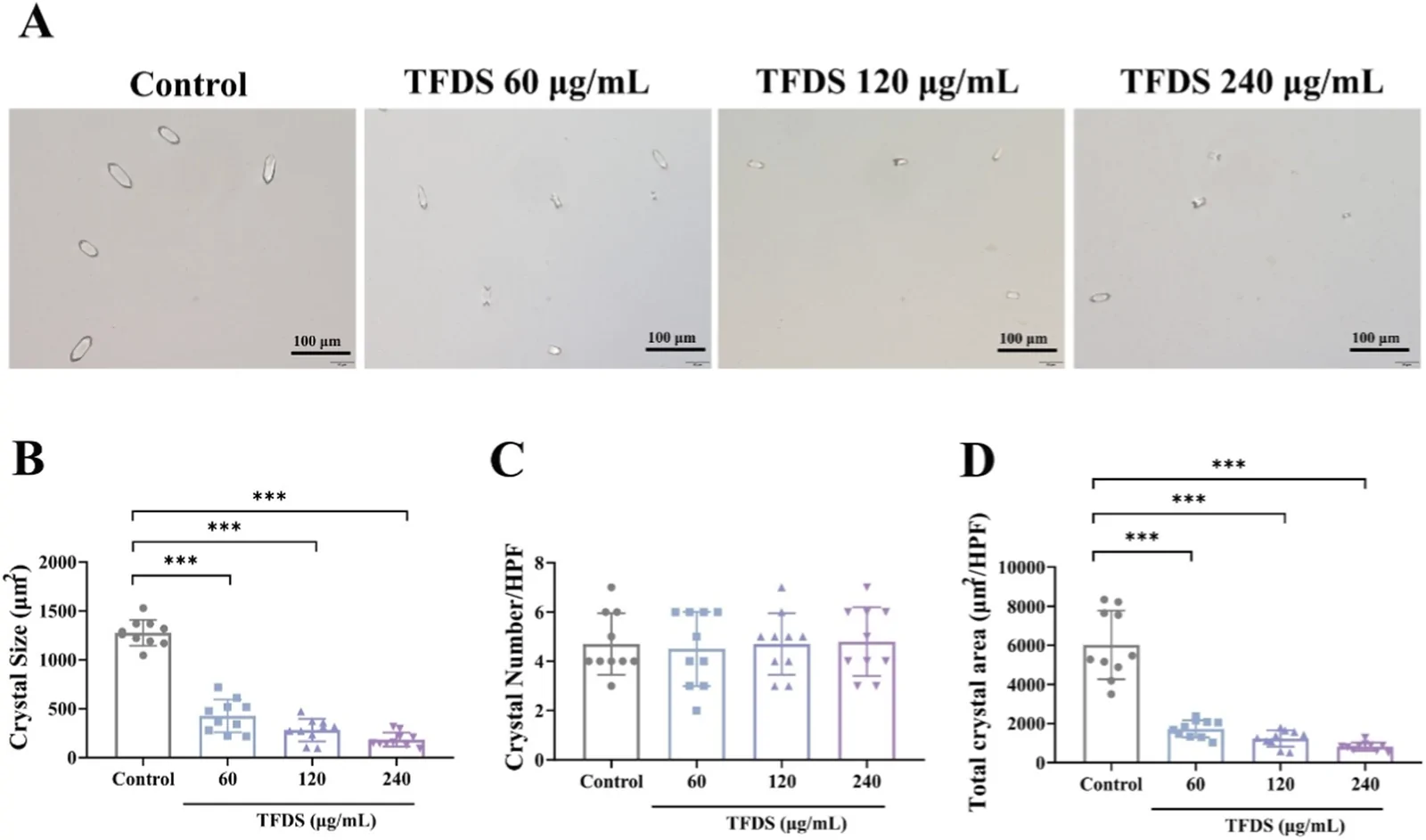
Inhibition of COM crystal growth by TFDS. Crystal growth assay was conducted on pre-formed crystals in the presence of DMSO (control) or TFDS (60–240 μg/mL). The crystallization buffer containing residual Ca^2+^ and C_2_O_4_^2-^ was retained, and TFDS was added directly to the buffer to assess its ability to inhibit further crystal growth in the presence of ions. **(A)** Representative images of crystal morphology under each condition. Scale bar, 100 µm. **(B)** Quantification of residual crystal size. **(C)** Quantification of residual crystal number. **(D)** Calculated residual total crystal area per HPF (Formula 1, Materials and Methods). Data are presented as mean ± SD. *p < 0.05, **p < 0.01, ***p < 0.001.

### 3.3 TFDS promotes the dissolution of COM crystals

To further investigate the crystal dissolution capacity of TFDS, a microscopic crystal dissolution assay was conducted. Following stable crystal formation, the crystallization buffer was replaced with physiological saline, and the effects of TFDS or a DMSO negative control were monitored microscopically (Figure 3A). Compared to the control, TFDS significantly reduced the average size across all tested concentrations (low, medium, and high) without markedly affecting the total number of crystals (Figures 3B,C). TFDS also decreased total crystal area per HPF across all doses, with a significant reduction observed at 240 μg/mL (Figure 3D). These results indicate that TFDS not only inhibits crystal nucleation and growth but also promotes the dissolution of established crystals.

**FIGURE 3:**
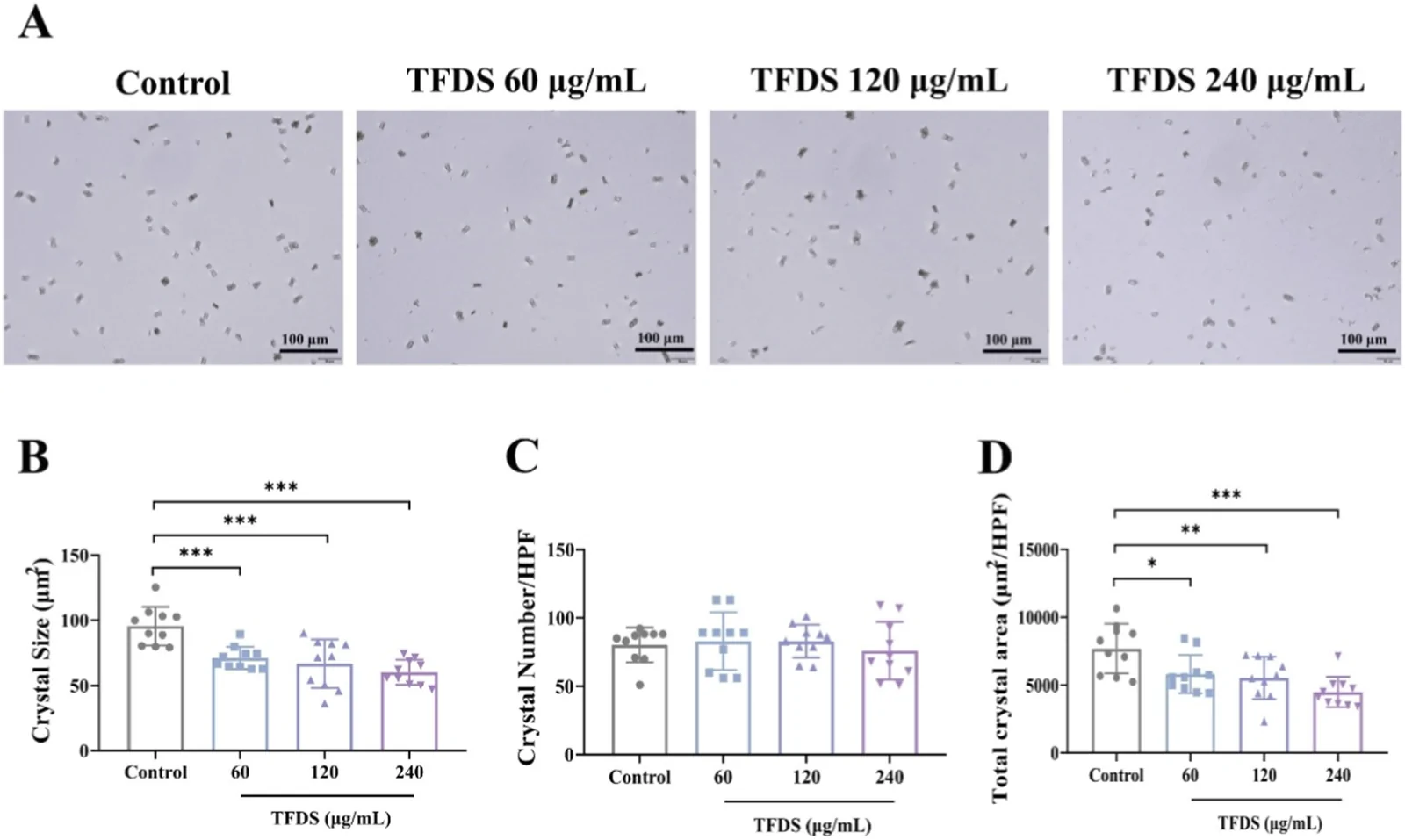
Dissolution of COM crystallization by TFDS. Crystal dissolution assay was performed by exposing established COM crystals to DMSO (control) or TFDS (60–240 μg/mL). In contrast to the growth assay, the ion-rich crystallization buffer was first removed, and the crystals were washed prior to the addition of TFDS in physiological saline. **(A)** Representative images of crystal morphology under each condition. Scale bar, 100 µm. **(B)** Quantification of crystal size. **(C)** Quantification of crystal number. **(D)** Calculated total crystal area per HPF (Formula 1, Materials and Methods). Data are presented as mean ± SD. *p < 0.05, **p < 0.01, ***p < 0.001.

### 3.4 TFDS prevents the adhesion of COM crystals to renal epithelial cell

Following crystal formation, the subsequent critical step in lithogenesis is the adhesion of these crystals to the renal tubular epithelium. To investigate the influence of TFDS on the detachment of COM crystals from the cell surface, graded concentrations of TFDS were introduced into the MDCK cell culture medium in the presence of COM crystals for 1 h incubation. Microscopic examination revealed a dramatic reduction in crystal attachment to the cell surface upon TFDS treatment (Figure 4A). Quantifications of the COM crystals also demonstrated that TFDS inhibited cellular attachment in a dose-dependent manner (Figure 4B). This suggests that TFDS may modify the crystal surface or cellular interface to prevent stable attachment.

**FIGURE 4:**
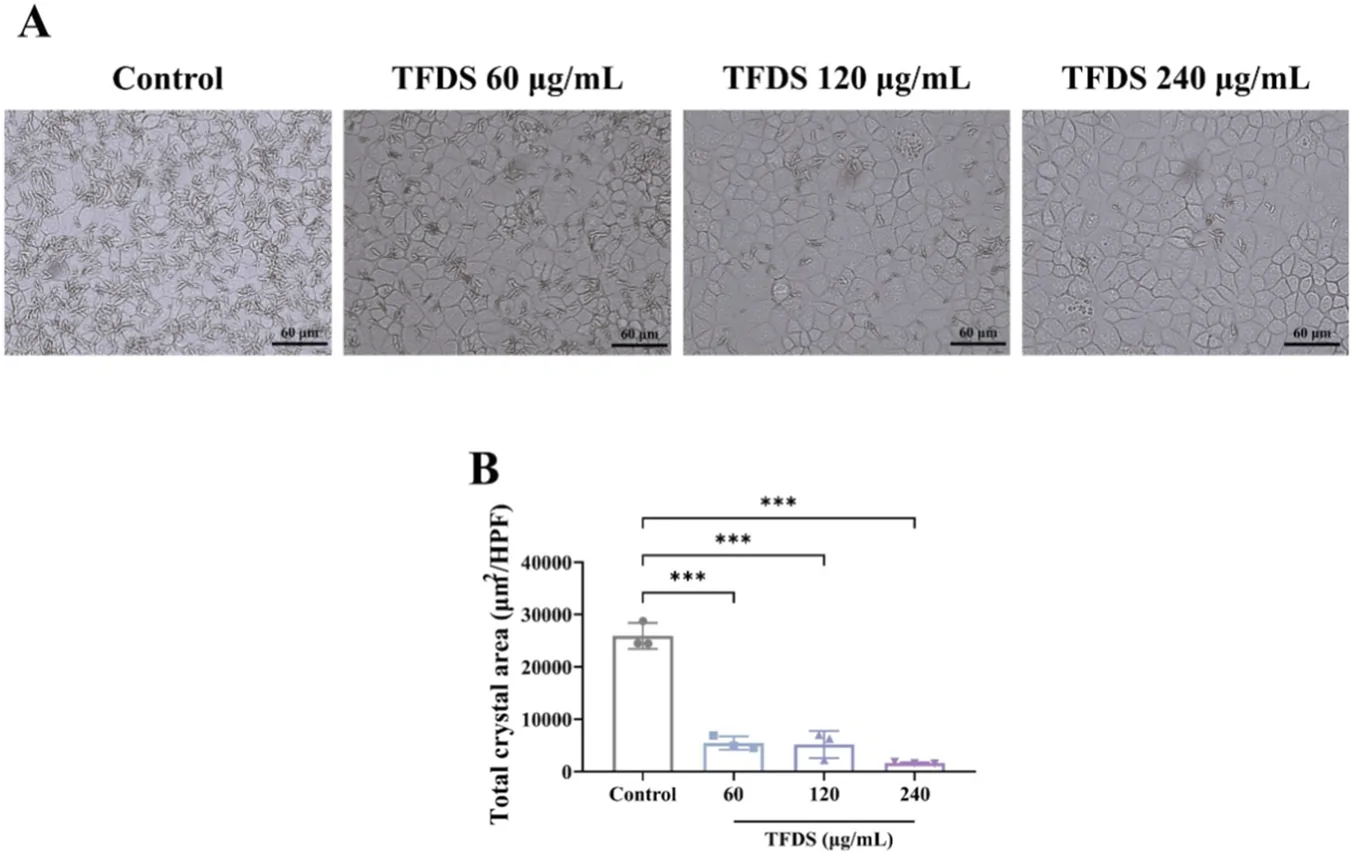
Detachment of COM crystals from the cell surface by TFDS. The crystal-cell adhesion assay was performed using MDCK cells treated with DMSO (control) or TFDS (60–240 μg/mL). **(A)** Representative micrographs showing the residual crystals that remained tightly adhered to the cell monolayer after PBS washes to remove unbound crystals. Scale bar, 60 µm. **(B)** Quantification of the adherent total crystal area per HPF (Formula 1, Materials and Methods). Data are presented as mean ± SD. *p < 0.05, **p < 0.01, ***p < 0.001.

### 3.5 TFDS inhibits crystal endocytosis in HK-2 cells

COM crystals are capable of internalizing into cells following their attachment to the cellular surface. To determine whether TFDS influence this internalization process, graded concentrations of TFDS were introduced into the HK-2 cell culture medium in the presence of FITC labeled COM crystals for 24 h incubation. The extent of crystal internalization was subsequently examined using flow cytometric analysis (Figure 5A). Treatment with TFDS resulted in a significant, dose dependent reduction in the percentage of FITC positive cells containing internalized COM crystals (Figure 5B). These results indicate that TFDS inhibits COM crystal internalization into the HK-2 cells.

**FIGURE 5:**
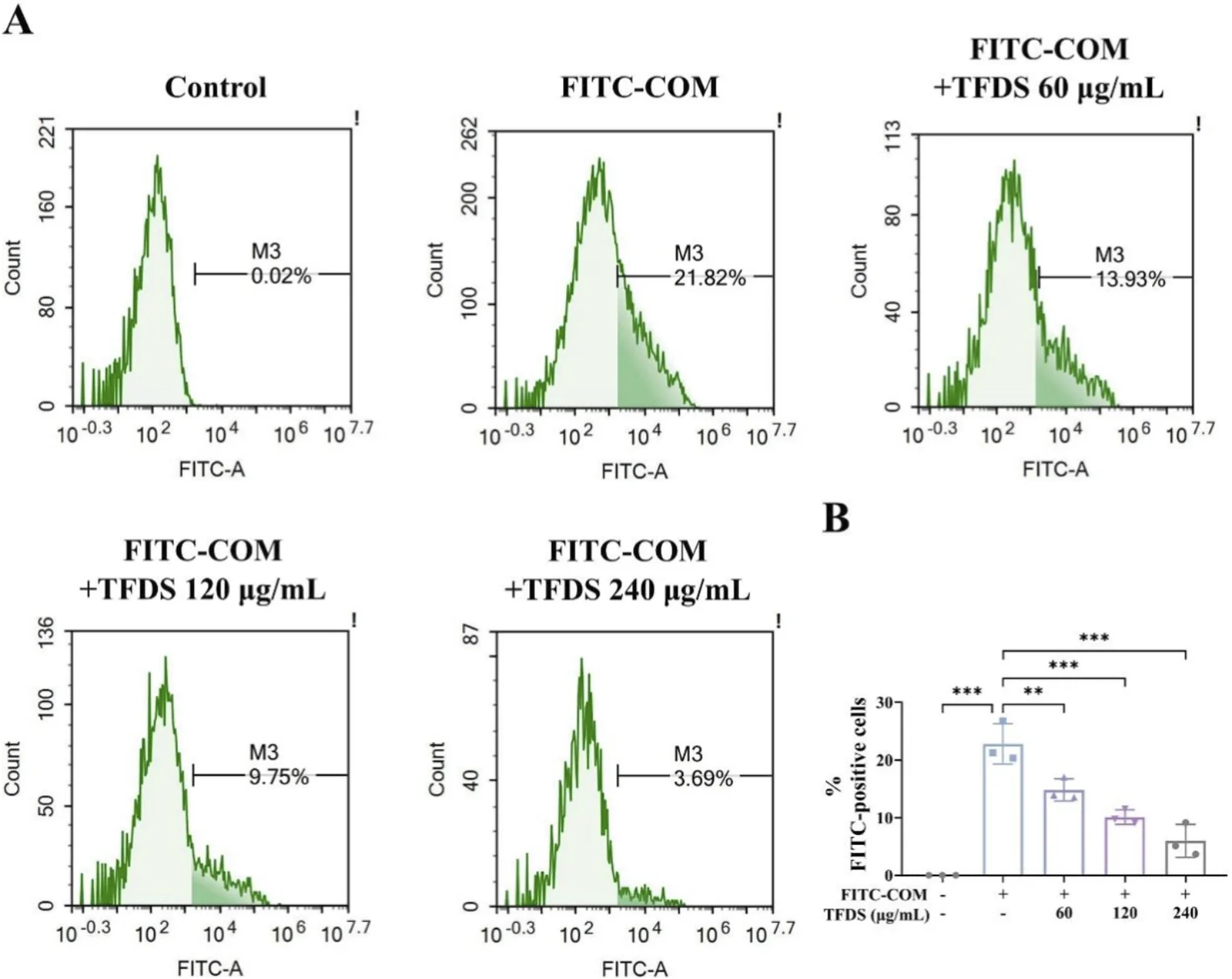
Inhibition of COM crystal internalization by TFDS. The crystal internalization assay was performed using HK-2 cells incubated with FITC-labeled COM crystals (FITC-COM) in the presence of DMSO (vehicle control) or TFDS (60–240 μg/mL). Background fluorescence was assessed using samples treated with unlabeled COM crystals. Non-internalized crystals were removed by trypsin-EDTA treatment prior to flow cytometry. **(A)** Representative histogram overlays showing FITC fluorescence intensity (corresponding to internalized crystals) under each treatment condition. **(B)** Percentage of FITC-positive cells with internalized crystals. Data are presented as mean ± SD. *p < 0.05, **p < 0.01, ***p < 0.001.

### 3.6 TFDS suppresses COM crystal-induced inflammatory response

Because crystal retention inevitably leads to cellular inflammation, we next evaluated the cytoprotective effects of TFDS (Kawase et al., 2024; Xu et al., 2024). To evaluate the effects of TFDS on crystal, IL-1β or LPS induced inflammation, HK-2 cells were exposed to COM crystals, IL-1β or LPS for 24 h. The expression levels of pro-inflammatory cytokines IL-6, IL-8, and TNF-α significantly increased in the cells treated with COM crystals, IL-1β or LPS compared with the control group (Figures 6A–I). After a 24 h treatment of TFDS, the expression levels of IL-6, IL-8, and TNF-α decreased in a dose-dependent manner when treated with COM crystals compared with the vehicle-treated model group (Figures 6A–C). However, when induced by IL-1β or LPS, TFDS treatment downregulated TNF-α, while the expression levels of IL-6 and IL-8 remained unchanged or slightly increased (Figures 6D–I). We hypothesize that this differential response is due to the complex, multi-component nature of TFDS. Distinct classes of flavonoids within the extract likely exert selective effects on diverging downstream signaling cascades depending on the primary inflammatory trigger. Moreover, exposure to COM crystals induced a significant elevation in the phosphorylated p65 (p-p65) to total p65 protein expression ratio relative to the control group (Figures 6J,K). Treatment with TFDS resulted in a dose-dependent reduction in the phosphorylation ratio of p65 relative to total p65 compared to the vehicle-treated model group (Figures 6J,K). Collectively, these results suggest that TFDS confers a protective, anti-inflammatory effect against COM crystal by modulating NF-κB signaling pathway.

**FIGURE 6:**
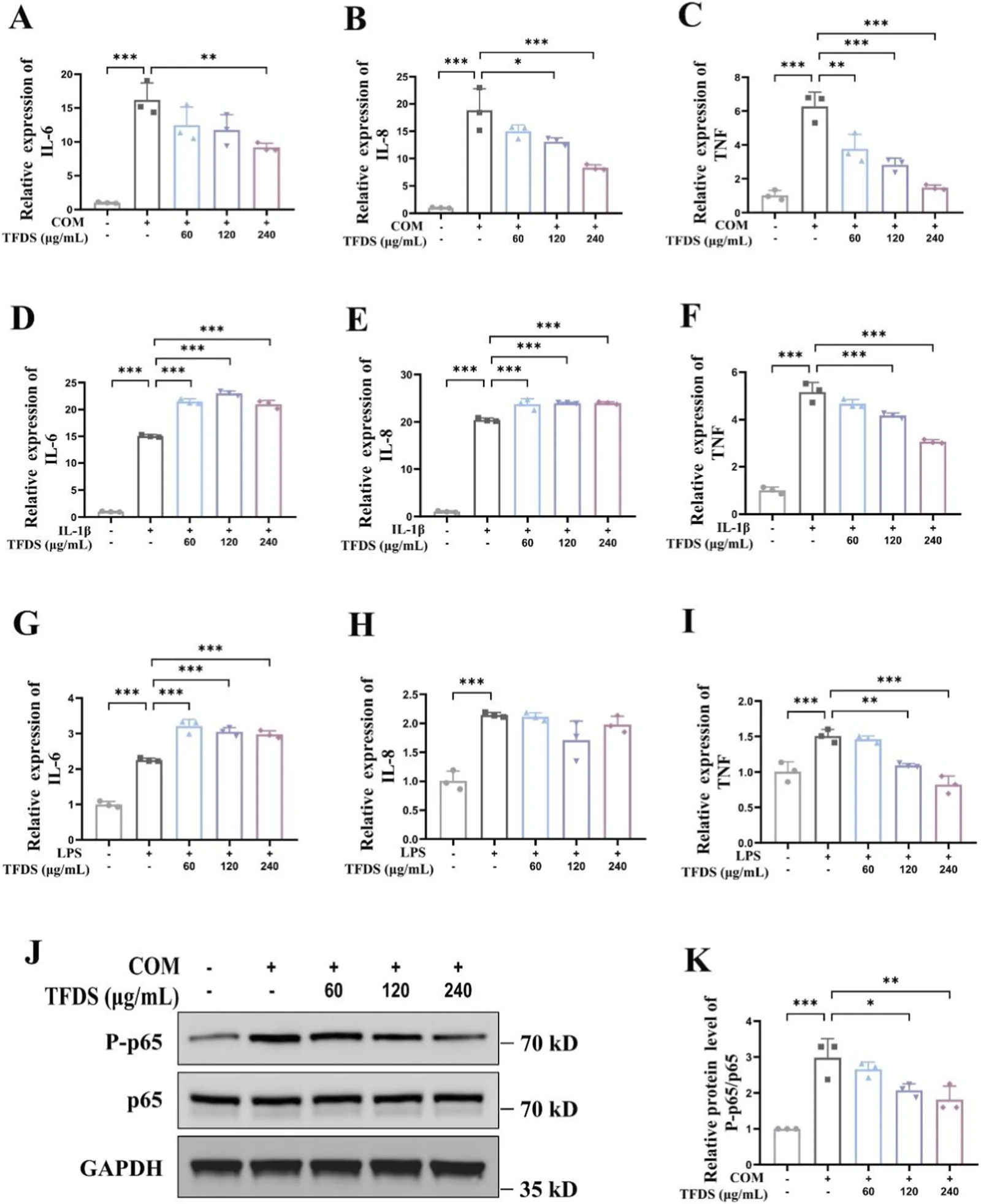
Inhibition of cellular inflammation by TFDS. HK-2 cells were co-treated with COM crystals, IL-1β or LPS and DMSO (control) or TFDS (60–240 μg/mL) for 24 h. Relative mRNA expression levels of *IL-6* **(A,D,G)**, *IL-8* **(B,E,H)**, and *TNF-*α **(C,F,I)** were determined by RT-qPCR, which were normalized to the mRNA expression levels of the geometric mean of *GAPDH* and *β-actin*. **(J)** Western blot indicated the protein expression levels of P-p65, p65, and GAPDH with indicated treatments. **(K)** Quantification of the Western blot bands in **(J)**. Data are presented as mean ± SD. *p < 0.05, **p < 0.01, ***p < 0.001.

### 3.7 TFDS ameliorates renal dysfunction in nephrolithiasis rats

To validate the *in vitro* findings within a physiological system, we evaluated the renal protective effects of TFDS in an ethylene glycol/ammonium chloride-induced rat model of nephrolithiasis. Experimental animals were administered either solvent or TFDS for a duration of 28 days subsequent to induction of renal calculi with ethylene glycol and ammonium chloride. To evaluate the impact of TFDS on renal function, both serum and urine samples were systematically collected and analyzed. BUN significantly increased in model group compared with the vehicle group. BUN decreased in both low (200 mg/kg) and high (400 mg/kg) dose TFDS groups compared with the model group, however the difference was significant only in high dose TFDS group but not in low dose TFDS group (Figure 7A). Serum creatinine significantly increased in model group compared with the vehicle group. Serum creatinine significantly decreased in high dose TFDS group compared with the model group (Figure 7B).

**FIGURE 7:**
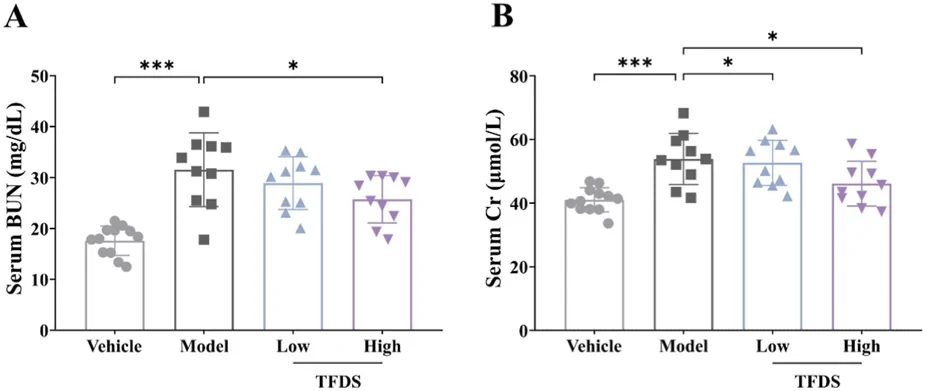
Effects of TFDS on serum biochemical parameters in a rat model of nephrolithiasis. Serum samples were collected from rats in the Control, Model, TFDS Low (200 mg/kg), and TFDS High (400 mg/kg) groups at the end of the experiment. Concentrations of **(A)** urea nitrogen, and **(B)** creatinine were determined using commercial assay kits. Data are presented as mean ± SD. *p < 0.05, **p < 0.01, ***p < 0.001.

Similar data were observed in urine samples. UUN (urine urea nitrogen) significantly increased in model group compared with the vehicle group. UUN significantly decreased in both low and high dose TFDS groups compared with the model group (Figure 8A). Urine creatinine also significantly decreased in both low and high dose TFDS groups compared with the model group (Figure 8B). During the experiments, we observed significant diuresis in the TFDS-treated animals. We hypothesize that this increased urine volume diluted the overall concentration of creatinine in the collected samples, leading to a lower measured concentration despite the concurrent recovery of baseline renal filtration function.

**FIGURE 8:**
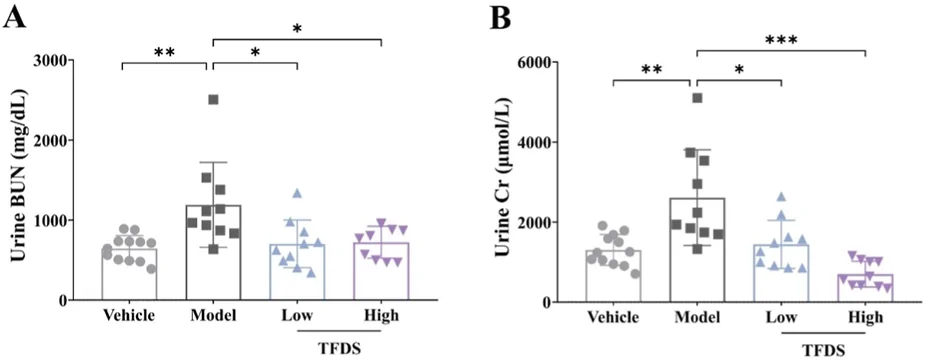
Effects of TFDS on urinary biochemical parameters in a rat model of nephrolithiasis. Urine samples were collected from rats in the Control, Model, TFDS Low (200 mg/kg), and TFDS High (400 mg/kg) groups at the end of the experiment. Concentrations of **(A)** urea nitrogen, and **(B)** creatinine were determined using commercial assay kits. Data are presented as mean ± SD. *p < 0.05, **p < 0.01, ***p < 0.001.

### 3.8 Ultrasonographic assessment confirms reduced renal crystal burden

Assessing the severity of renal calculi in live rats presents considerable challenges. Therefore, a noninvasive imaging approach was employed. Ultrasound examination of the kidneys was conducted prior to animal sacrifice to evaluate crystals formation. Specifically, ultrasonographic assessments were performed on day 28 following administration of either solvent or TFDS (Figure 9A). Renal echogenicity was measured in vertical and horizontal views to assess the severity of crystal deposition. The values of both the vertical and horizontal view intensities significantly increased in the model group compared with the vehicle group (Figures 9B,D). Vertical view intensities were significantly decreased in both low (200 mg/kg) and high (400 mg/kg) dose TFDS groups compared with the model group (Figure 9B). Horizontal view intensity was significantly decreased in high dose TFDS group, which was also decreased in low dose TFDS group, although the differences were not significant (Figure 9D). The differences of renal long axis between model group and TFDS groups were not significant in either vertical or horizontal view (Figures 9C,E), however both vertical and horizontal long axis of model group slightly increased compared with the vehicle group, then decreased slightly after treatment with either low or high dose of TFDS.

**FIGURE 9:**
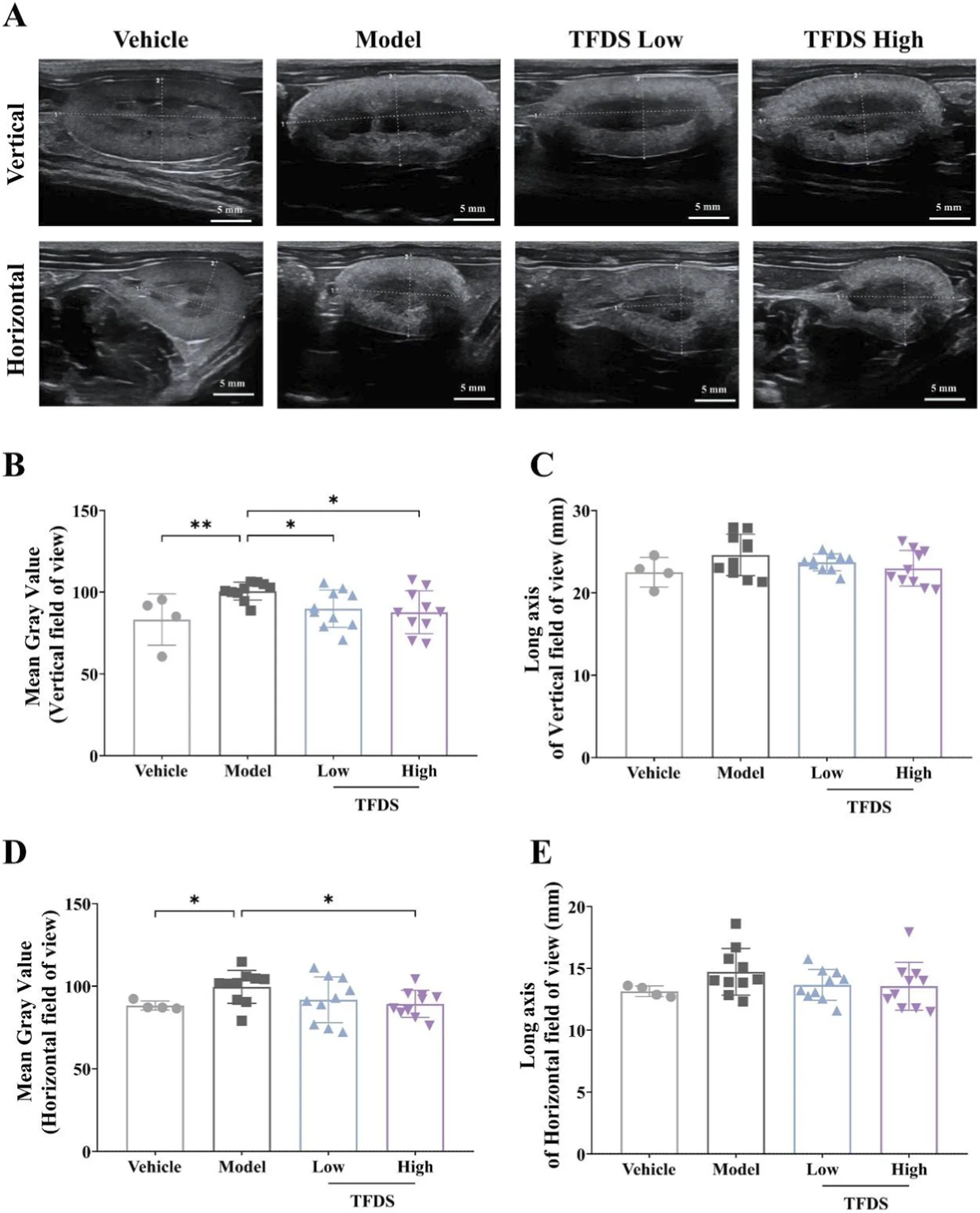
TFDS ameliorates renal ultrasonographic features in a rat model of nephrolithiasis. Renal ultrasound examination was performed in rats in the Control, Model, TFDS Low (200 mg/kg), and TFDS High (400 mg/kg) groups at the end of the experiment. **(A)** Representative B-mode ultrasound images of the kidneys, scale bar, 5 mm. Quantitative analyses of **(B)** mean gray value (echogenicity) and **(C)** long-axis length in the vertical field of view, and **(D)** mean gray value and **(E)** long-axis length in the horizontal field of view. Data are presented as mean ± SD. *p < 0.05, **p < 0.01, ***p < 0.001.

### 3.9 Histopathological findings of renal protection

Histological analysis was performed to determine the capacity of TFDS to preventing renal crystal retention and preserving tissue architecture. The Von Kossa staining and quantification showed a significant reduction in both the quantity and area of COM crystallization in high (400 mg/kg) dose TFDS group compared with model group (Figures 10A–C). Moreover the H&E staining results showed that less renal tubular degeneration and necrosis, basophilic change of renal tubules, interstitial inflammatory cell infiltration, and renal tubular dilation in the kidneys of rats treated with TFDS compared with the model group (Figure 10A). These results provide additional evidence for the anti-lithogenic properties of TFDS, consistent with observations from biochemical assays and ultrasound evaluations.

**FIGURE 10:**
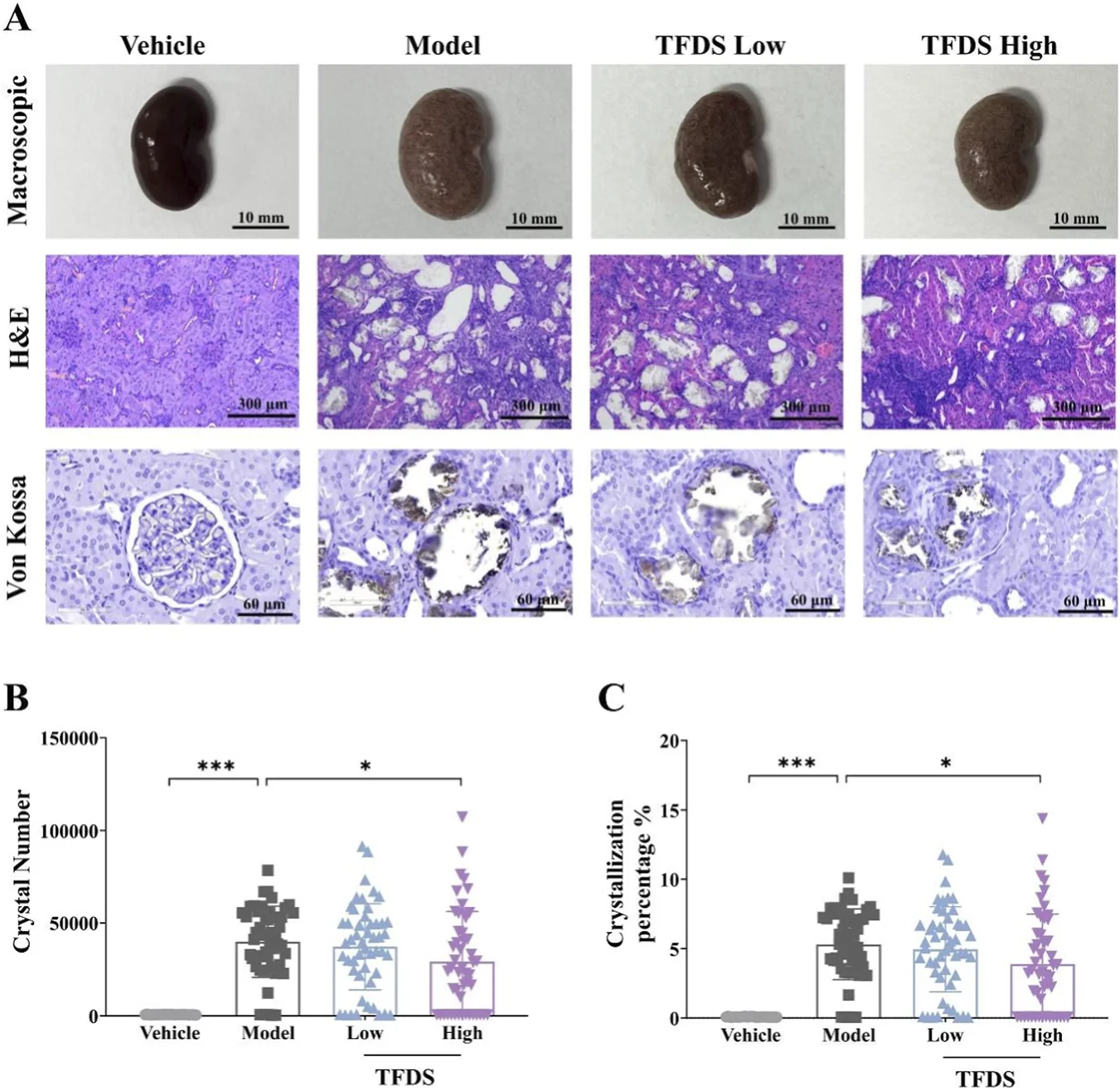
TFDS mitigates renal histopathological injury and crystal deposition in a rat model of nephrolithiasis. Kidney tissues were harvested from rats in the Control, Model, TFDS Low (200 mg/kg), and TFDS High (400 mg/kg) groups at the end of the experiment. **(A)** Representative macroscopic appearance of the kidney (scale bar, 10 mm), H&E staining (scale bar, 300 µm), and von Kossa staining (scale bar, 60 µm) of kidney tissue sections. Quantitative analyses of **(B)** crystal number per high-power field (HPF) and **(C)** crystallization area percentage. Data are presented as mean ± SD. *p < 0.05, **p < 0.01, ***p < 0.001.

## 4 Discussion

Nephrolithiasis imposes a substantial clinical and socioeconomic burden, disproportionately affecting young and middle-aged populations (Allam, 2024). Despite the rising global prevalence and high recurrence rates, surgical interventions carry risks of hemorrhage and organ damage, rendering certain patient populations unsuitable candidates for surgery. Moreover, surgery is typically not indicated for patients presenting with small calculi. Consequently, the development of novel therapeutic agents capable of attenuating lithogenesis and mitigating renal damage is of critical clinical significance.

*Desmodium styracifolium* total flavonoids capsule has emerged as a clinically significant intervention for urolithiasis, since its market approval in China in 2022. As a traditional Chinese medicine pharmacotherapy for nephrolithiasis, the therapeutic efficacy of *D. styracifolium* has been substantiated by several foundational studies. Research has demonstrated that *D. styracifolium* extracts exert potent antilithic effects in rat models by inducing diuresis, reversing hypercalciuria and hypocitraturia, and mitigating renal oxidative stress (Hirayama et al., 1993; Mi et al., 2012; Xiang et al., 2015). However, these historical studies largely relied on crude extracts and focused on systemic *in vivo* outcomes. A significant gap remains in understanding the precise *in vitro* biophysical and cellular mechanisms of the plant’s primary standardized bioactive constituent, the total flavonoids (*D. styracifolium* total flavonoids, TFDS).

As the primary bioactive constituent of *D. styracifolium*, TFDS serve as the principal therapeutic agent in the *D. styracifolium* total flavonoids capsules. While existing literature has documented the efficacy of TFDS in managing nephrolithiasis, attributing its effects to antioxidant activity, anti-inflammatory responses, urinary alkalization, and the modulation of lithogenic mineral excretion in rat kidney calculi models, the specific biochemical mechanisms underlying these inhibitory effects remain poorly defined (Zhou et al., 2018). While, other related compounds like diosmin have been reported to exhibit inhibitory effects on crystal formation and growth *in vitro* (Khamchun et al., 2021). Expanding on this conceptual framework, we conducted a systematic analysis of the effects of TFDS on calcium oxalate kinetics, utilizing *in vitro* assays to delineate its inhibitory efficacy. Our findings demonstrate that TFDS significantly attenuates crystal-cell adhesion and subsequent cellular internalization. Moreover, our results demonstrate that TFDS downregulates critical inflammatory factors and mitigates cellular inflammation, indicating its potential role in attenuating inflammation within the kidney. To the best of our knowledge, these properties are unprecedented in the literature, demonstrating that TFDS exerts an inhibitory influence throughout the entire process—spanning from crystal formation to its eventual internalization into cells. Collectively, these results offer a series of evidence for the clinical use of *D. styracifolium* total flavonoids capsules in the management of nephrolithiasis. While our data clearly demonstrate the inhibitory effects of TFDS on crystal formation and inflammation, a limitation of the current study is the absence of advanced structural and chemical analyses, such as X-ray crystallography or the investigation of potential TFDS-oxalate conjugates. Future studies employing these biophysical techniques are warranted to fully map the precise physical mode of action underlying crystal inhibition.

In an *in vivo* rat model of nephrolithiasis, TFDS significantly inhibits kidney calculi formation, showing a strong correlation with its previously observed *in vitro* inhibition of crystallization properties. This nephrolithiasis rat model is extensively utilized to assess the efficacy of pharmacological agents (Chen et al., 2025; Elgendy et al., 2025). The efficacy of TFDS in reducing kidney calculi retention appears to stem from its dual ability to inhibit crystal-cell adhesion and cellular internalization, thereby facilitating renal functional recovery. In this model, biochemical analysis revealed that urea nitrogen and creatinine exhibited a strong correlation with the incidence of nephrolithiasis. Notably, we employed ultrasound imaging as an innovative modality for the qualitative and quantitative assessment of the therapeutic efficacy of TFDS, which is a methodology previously undocumented in relevant literature. Our data demonstrated a high degree of correlation between ultrasonographic data and histopathological findings, both confirming that TFDS significantly attenuates renal crystal retention. This consistency underscores the diagnostic precision of ultrasonography as a high-fidelity, non-invasive detection method for monitoring renal calculi burden. Furthermore, ultrasonography enables a relatively accurate assessment of the severity of renal stone formation and retention without necessitating animal sacrifice (Sakthikarthikeyan et al., 2025). It facilitates longitudinal monitoring of the dynamic progression of nephrolithiasis across various stages, including model induction, group allocation, and throughout the treatment period (Iacobellis et al., 2019).

In summary, this study demonstrates that TFDS effectively mitigates the progression of nephrolithiasis by inhibiting crystal formation and deposition. These *in vitro* and *in vivo* findings provide a mechanistic framework for the therapeutic efficacy of TFDS capsules in the clinical management of nephrolithiasis. While TFDS is a concentrated extract of *D. styracifolium*, it remains a complex multi-component fraction. The importance of identifying single active constituent is underscored by existing literature. For instance, schaftoside has been reported to modulate key pathological processes, including the suppression of inflammatory cytokine cascades, the inhibition of apoptotic signaling, and the attenuation of oxidative stress (Zhou et al., 2019; Liu et al., 2020; Dang et al., 2021; Zhang et al., 2022; Lu et al., 2024). Schaftoside has also been shown to alleviate acute liver injury by modulating the ferroptosis pathway (Yu et al., 2024). Furthermore, styracifoline, another isolate from *D. styracifolium*, has demonstrated inhibitory activity against proteins associated with diabetes and thrombosis (Tran et al., 2021). Future studies are therefore investigated to determine which primary single component drives the observed antilithogenic and renal protective effects.

## 5 Conclusion

In conclusion, this study elucidates the multi-faceted inhibitory effects by which TFDS attenuates nephrolithiasis, specifically through reducing COM crystallization and inflammation. We demonstrate that TFDS exerts an inhibitory effect throughout the lithogenic cascade, spanning crystal nucleation, growth, dissolution, cellular adhesion, and subsequent endocytosis. Beyond physical inhibition, TFDS treatment significantly mitigates COM-induced inflammatory responses within renal tissues. The therapeutic efficacy was further substantiated by longitudinal ultrasonographic monitoring and histopathological analysis, which revealed a marked reduction in renal crystal retention. Collectively, these findings provide a perspective on the functions and actions of TFDS for the application of *D. styracifolium* total flavonoids capsules in the management of nephrolithiasis.

## Data Availability

The original contributions presented in the study are included in the article/supplementary material, further inquiries can be directed to the corresponding authors.
